# Exercise Crystal: simulations that drive National  IHR Focal Point capacity-strengthening

**DOI:** 10.5365/wpsar.2025.16.2.1240

**Published:** 2025-06-04

**Authors:** Laura Goddard, Qiu Yi Khut, Gina Samaan

**Affiliations:** a WHO Health Emergencies Programme, World Health Organization Regional Office for the Western Pacific, Manila, Philippines.

## Abstract

The International Health Regulations (2005; IHR) require States Parties to designate a National IHR Focal Point (NFP; i.e. a national centre) to ensure timely communications with the World Health Organization (WHO) about all events that may constitute a public health emergency of international concern and, following recent amendments, to designate a National IHR Authority to coordinate IHR (2005) implementation within the Parties. Since 2008, the WHO Regional Office for the Western Pacific has been running an annual simulation exercise, known as the IHR Exercise Crystal, to test and strengthen NFP functionality. This study analyses NFP performance during the IHR Exercise Crystal over a 16-year period (2008–2024, excluding 2009) to inform Member States’ planning for NFP capacity-strengthening in the context of the recent IHR (2005) amendments. Data collected about NFP performance during these exercises were analysed using descriptive statistics across six key NFP performance indicators. Key findings show that the proportion of NFPs that are accessible via e-mail is consistently high (mean: 99%), but there is suboptimal NFP accessibility via telephone (mean: 64%). The proportion of NFPs participating in tele- and videoconferencing during the exercise improved over time (mean: 73%), as did the proportions of NFPs notifying WHO of simulated events (mean: 80%) and contributing information to the Event Information Site for NFPs (mean: 77%). The proportion engaging in multisectoral communication remained variable, with no clear trend (mean: 73%). These results demonstrate that significant progress has been made in strengthening NFP functionality, but there are opportunities for further improvement, particularly in the areas of telephone accessibility and multisectoral coordination. It is critical that States Parties continue strengthening and testing NFP functionality through simulation exercises and other capacity-building activities to ensure effective IHR (2005) implementation. Furthermore, States Parties should develop, test and maintain up-to-date standard operating procedures to support the clear demarcation of roles and responsibilities between the NFP and the National IHR Authority.

The International Health Regulations (2005; IHR) define countries’ rights and obligations in handling public health events and emergencies that have the potential to cross borders. (*1*) Signatory States Parties commit to developing core capacities for public health emergencies and, under Article 4, are required to designate or establish a National IHR Focal Point (NFP; i.e. a national centre) to communicate with the World Health Organization (WHO). The NFP must be accessible at all times to communicate with WHO IHR Contact Points and is responsible for sending information to WHO on behalf of States Parties, as well as disseminating information to, and consolidating input from, relevant sectors of the Party’s administration. (*1*, *2*) In the case of territories and areas, States Parties may establish IHR Contact Points specific to each territory or area for the purpose of IHR (2005) communications, although this is not mandatory under the IHR (2005).

IHR (2005) communications and the role of the NFP are central to the Regulations and to global health security. In the recent amendments to the Regulations, which were approved by Member States in June 2024, the requirement to designate or establish an NFP has remained unchanged. (*3*) However, States Parties must now designate or establish a National IHR Authority, which may be the same entity as an NFP, or a different entity, to coordinate implementation of the Regulations within the State Party. (*3*) Therefore, in some countries the designation of a National IHR Authority may have an impact on current NFP practices or operations.

In the WHO Western Pacific Region, IHR (2005) implementation is supported through the Asia Pacific Health Security Action Framework (APHSAF). (*4*) Progress in implementing the IHR (2005), including the NFP and other core capacities, is assessed and monitored through the mandatory States Parties Self-Assessment Annual Report (SPAR) and voluntary assessments, including Joint External Evaluations, simulation exercises, and intra- and after-action reviews. (*1*, *5*) These assessments help to identify strengths and areas for improvement and then translate them into priority actions as part of national planning to build a country’s capacities. (*6*)

In the Western Pacific Region, the functioning of NFP capacities is routinely assessed through an annual simulation exercise organized by the WHO Regional Office for the Western Pacific. IHR Exercise Crystal has been running since 2008 and aims to test IHR (2005) communication channels and familiarize NFPs and WHO staff with the IHR communication system. (*7*) Findings from the exercise can inform actions needed to strengthen NFP functionality and should be triangulated with other assessments to better understand the capacity level of countries and to implement priority actions at the national and subnational levels. (*5*)

This analysis describes the performance of IHR NFPs and Contact Points in the Western Pacific Region during IHR Exercise Crystal over time, with the aim of informing Member States’ planning as they prepare for implementing the IHR (2005) amendments.

## Methods

### Study design

We conducted a descriptive analysis of IHR NFP and Contact Point performance during IHR Exercise Crystal over a 16-year period (2008–2024, excluding 2009).

### Study population and setting

The Regional Office works with health authorities from 37 countries and areas, of which 22 are Pacific island countries and areas, totalling more than one quarter of the world’s population. (*8*) The Region is very diverse; significant variations exist in Member States’ geography, demographics, health systems and services, disease burden and disaster risk profiles. (*9*-*11*) There are, however, many common challenges and approaches that countries and areas in the Region share, including a common approach to IHR (2005) implementation and capacity development through APHSAF. (*4*)

### Data sources and analysis

Data about NFP performance during IHR Exercise Crystal were extracted from reports and monitoring data sets for the years 2008–2024. Monitoring and evaluation data have been collected each year of IHR Exercise Crystal to measure IHR NFP and Contact Point performance against the exercise’s objectives (**Table 1**); however, when monitoring data sets were not available – for 2008, 2010, 2019 and 2021 – only exercise reports were used.

**Table 1:** IHR Exercise Crystal objectives, by year, 2008–2024

Objetive	Year																
	2008	2009	2010	2011	2012	2013	2014	2015	2016	2017	2018	2019	2020	2021	2022	2023	2024
**Validate the accessibility of NFPs using their registered contact details**	**X**		**X**	**X**	**X**	**X**	**X**	**X**	**X**	**X**	**X**	**X**	**X**	**X**	**X**	**X**	**X**
**Practise and test the IHR (2005) notification process**	**NA**		**X**	**X**	**X**	**X**	**X**	**X**	**X**	**X**	**X**	**X**	**X**	**X**	**X**	**X**	**X**
**Assess multisectoral communication between NFPs and national counterparts**	**NA**		**NA**	**NA**	**NA**	**NA**	**X^a^**	**NA**	**X^b^**	**X^c^**	**X**	**X**	**X**	**X**	**X**	**X**	**X**
**Improve NFPs’ understanding of the IHR (2005) communication system**	**NA**		**NA**	**NA**	**NA**	**NA**	**NA**	**X**	**X**	**X**	**X**	**X**	**X**	**X**	**X**	**X**	**X**
**Test whether NFPs use tele- or videoconferencing**	**NA**		**X^d^**	**X**	**X**	**NA**	**NA**	**X^e^**	**X^d^**	**X**	**X**	**X**	**X**	**X**	**X**	**X**	**X**
**Additional objectives**	**^f, g^**		**^f^**	**NA**	**NA**	**^h^**	**NA**	**^i^**	**^j, k^**	**^i^**	**NA**	**NA**	**^l^**	**^l^**	**^l^**	**NA**	**NA**

IHR: International Health Regulations (2005); NA: not applicable; NFP: National Focal Point.

^a^ The objective was to facilitate communication and collaboration between NFPs and emergency contacts for the International Food Safety Authorities Network   (INFOSAN), a joint initiative of the Food and Agriculture Organization of the United Nations and WHO, during a foodborne illness emergency.

^b^ The objective was to examine protocols for NFPs working with non-health actors, particularly national disaster management agencies.

^c^ The objective was to improve collaboration with other agencies.

^d^ This was not listed as an objective but was still tested.

^e^ Videoconferencing was introduced to IHR Exercise Crystal in 2015.

^f^ An additional objective aimed to test the WHO guide on the IHR (2005) Communications and Duty Officer system (internal document).

^g^ The objective was to validate the IHR (2005) verification process between Member States and WHO in response to a public health emergency of international   concern.

^h^ This focused on improving the engagement of WHO country offices to facilitate communication between NFPs and WHO IHR Contact Points.

^i^ The aim was for NFPs to practise the use of IHR (2005) principles and obligations, and to evaluate NFPs’ understanding of these.

^j^ The objective was to familiarize participants with the IHR (2005) Emergency Committees.

^k^ This aimed to test NFPs’ ability to log on and use the Event Information Site platform.

^l^ A structured scenario was used to explore key issues to identify strengths and areas needing improvement.

*Source:* Adapted from Table 2 in Li and Li. (*12*)

The study used descriptive statistics to summarize and describe the performance of IHR NFPs and Contact Points in the Western Pacific Region during the exercise for six key variables relating to NFP functions (**Table 2**). For each variable, the number and percentage of countries meeting the criteria were calculated for each year, and the mean was calculated for all years. These variables assess whether the IHR NFP or Contact Point is available by e-mail or telephone, or through teleconferencing or videoconferencing; whether they notified WHO about the public health event during the exercise; whether they prepared information to share via the Event Information Site (EIS) for NFPs; and whether they engaged with other stakeholders during the exercise.

**Table 2:** IHR Exercise Crystal definitions of variables

Variable	Definition
E-mail	The IHR NFP or Contact Point could be successfully contacted by e-mail during the exercise, either through registered contact details or alternative details that had been provided.
Telephone	The IHR NFP or Contact Point could be successfully contacted by telephone during the exercise, either through registered contact details or alternative details that had been provided.
Tele- or videoconference	The IHR NFP or Contact Point could successfully join a tele- or videoconference call, held at the beginning or conclusion of the exercise.
Notification	The IHR NFP or Contact Point e-mailed the WHO IHR Contact Point to notify the public health event during the exercise.
EIS posting^a^	The IHR NFP or Contact Point e-mailed the WHO IHR Contact Point to share information about the public health event during the exercise for inclusion in an EIS posting.
Multisectoral communication	During the exercise, the IHR NFP or Contact Point contacted either the exercise Simulator^b^ instead of another agency or sector within their country, or a specifically nominated agency, during the exercise.

EIS: Event Information Site; IHR: International Health Regulations (2005); NFP: National Focal Point; WHO: World Health Organization.

^a^ An EIS posting is a summary of a public health event and an assessment of its risk that is published to the EIS platform by WHO and accessible to all States Parties. Each EIS posting contains the following elements: core event details, WHO IHR Contact Point details, IHR (2005) Annex 2 criteria, situation summary, public health response, a WHO risk assessment and WHO’s recommendations. The EIS process involves WHO assessing whether information about public health events should be posted to the EIS platform, based on certain criteria, and if the criteria are met, initiating an EIS posting in consultation with Member States. However, in practice, some Member States also initiate EIS postings in consultation with WHO, and these are subject to the same WHO criteria and assessment.

^b^ The Simulator is a role created for the purposes of IHR Exercise Crystal and is played by designated staff in the WHO Regional Office for the Western Pacific during the exercise. Participating countries and areas may contact the Simulator instead of a real government department, other agency or expert that they would like to interact with as part of the exercise scenario. The Simulator only responds to e-mails from participating countries and areas; contact is not initiated by the Simulator.

## Results

**Fig. 1:**
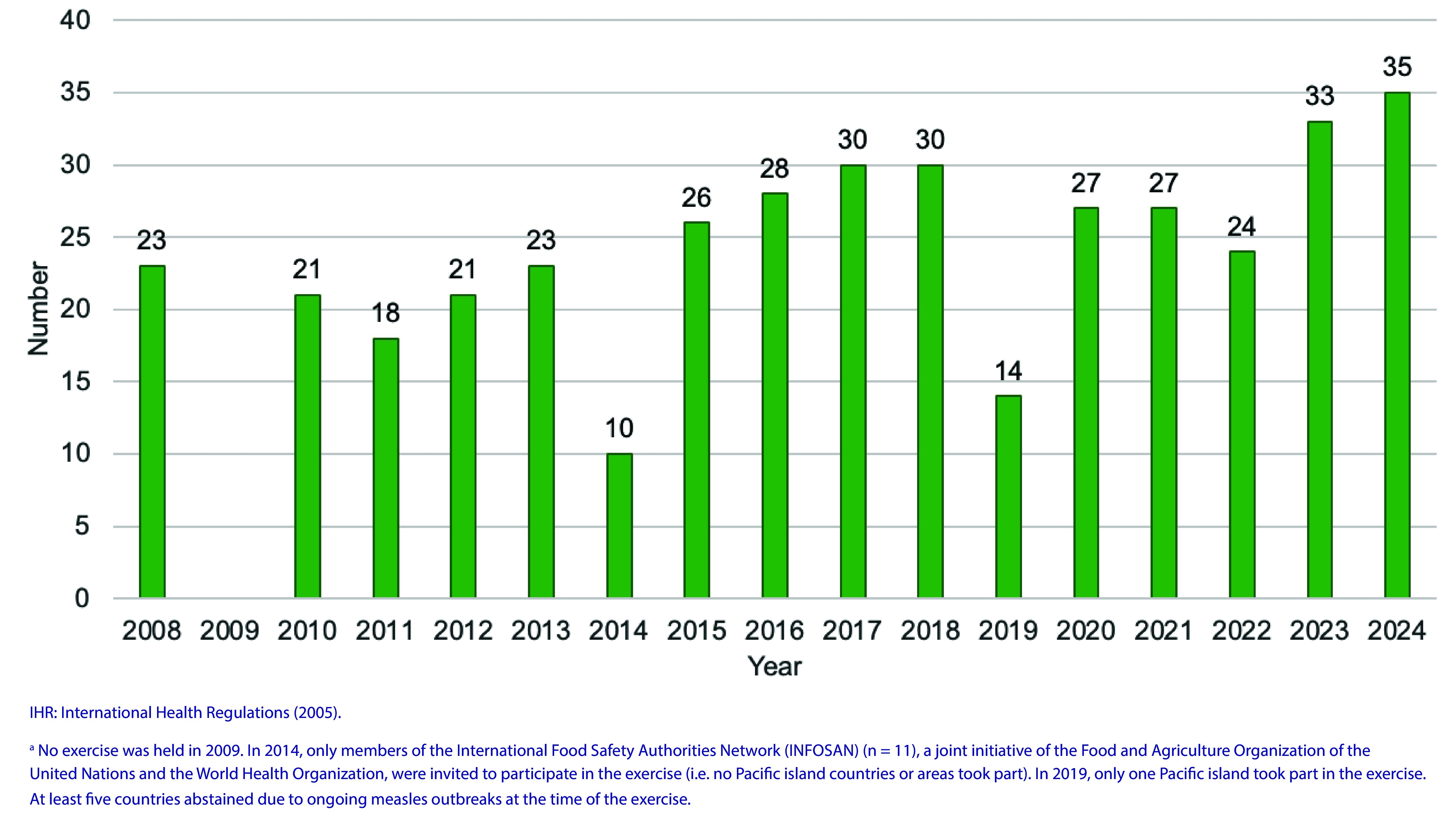
Number of countries and areas participating in IHR Exercise Crystal, by year, 2008–2024^a^

Between 2008 and 2024, IHR Exercise Crystal was held 16 times. No exercise was held in 2009; however, during 2009, NFPs communicated frequently with the WHO IHR Contact Point on pandemic influenza A(H1N1), which was a public health emergency of international concern. Additionally, in 2014, an exercise was held with the International Food Safety Authorities Network (INFOSAN), jointly governed by the Food and Agriculture Organization of the United Nations and WHO, which was limited to the then-11 INFOSAN member countries. In 2019, at least five countries were unable to participate in the exercise due to measles outbreaks, leading to a lower participation rate that year. Eleven of the 16 exercise scenarios were related to outbreaks of respiratory viruses (**Table 3**). From 2008 to 2015 (excluding 2014, only States Parties (*n* = 27) to the IHR (2005) in the Region were invited to participate, with an average of 22 participating countries and areas (range: 18–26) taking part each year. From 2016 onwards, all 37 countries and areas in the Region were invited to participate, with an average of 28 (range: 14–35) taking part each year (**Fig. 1**). Mean participation for the period 2008–2024 (including 2014) was 78% (**Table 3**).

[**insert** Figure 1]

**Table 3:** Summary of the performance of participating countries and areas during IHR Exercise Crystal, by year, 2008–2024

Year	Scenario	No. (%) of participants^a^	Variable^b^					
			E-mail	Telephone	Tele- or video-conferencing	IHR (2005) notification^c^	EIS posting^c^	Multisectoral communication
**2008**	**Disease X outbreak**	**23 (85)**	**19 (83)**	**13 (57)**	**–^d^**	**–**	**–**	**–**
**2009**	**-**	**-**	**-**	**-**	**-**	**-**	**-**	**-**
**2010**	**Disease X outbreak**	**21 (78)**	**21 (100)**	**12 (57)**	**15 (71)**	**–**	**–**	**–**
**2011**	**Severe acute respiratory illness outbreak**	**18 (67)**	**17 (94)**	**9 (50)**	**16 (89)**	**5 (28)**	**9 (50)**	**–**
**2012**	**Influenza-like illness outbreak**	**21 (78)**	**21 (100)**	**18 (86)**	**17 (81)**	**15 (71)**	**12 (57)**	**–**
**2013**	**Severe acute respiratory illness outbreak**	**23 (85)**	**23 (100)**	**15 (65)**	**–**	**18 (78)**	**17 (74)**	**–**
**2014**	Verocytotoxin-producing ***Escherichia coli*** outbreak	**10 (91)**	**10 (100)**	**6 (60)**	**–**	**10 (100)**	**10 (100)**	**10 (100)**
**2015**	**Novel influenza outbreak**	**26 (96)**	**26 (100)**	**21 (81)**	**10 (38)**	**21 (81)**	**20 (77)**	**–**
**2016**	**Novel coronavirus outbreak**	**28 (76)**	**28 (100)**	**8 (29)**	**8 (29)**	**22 (79)**	**21 (75)**	**18 (64)**
**2017**	**Novel influenza outbreak in cats, with human infections**	**30 (81)**	**30 (100)**	**26 (87)**	**18 (60)**	**26 (87)**	**24 (80)**	**24 (80)**
**2018**	Novel ***Francisella tularensis*** outbreak (deliberate use of a biological agent)	**30 (81)**	**30 (100)**	**25 (83)**	**20 (67)**	**26 (87)**	**24 (80)**	**16 (53)**
**2019**	**Novel influenza outbreak**	**14 (38)**	**14 (100)**	**–**	**–**	**14 (100)**	**14 (100)**	**–**
**2020**	**Potential adverse effects following immunization with a vaccine for a novel respiratory virus**	**27 (73)**	**27 (100)**	**20 (74)**	**17 (63)**	**25 (93)**	**21 (78)**	**20 (74)**
**2021**	**Novel influenza outbreak**	**27 (73)**	**27 (100)**	**17 (63)**	**23 (85)**	**21 (78)**	**19 (70)**	**22 (81)**
**2022**	**Novel influenza outbreak**	**24 (65)**	**24 (100)**	**12 (50)**	**23 (96)**	**23 (96)**	**24 (100)**	**21 (88)**
**2023**	**Radiological event**	**33 (89)**	**33 (100)**	**20 (61)**	**31 (94)**	**30 (91)**	**14 (42)**	**17 (52)**
**2024**	**Emerging arbovirus outbreak (Oropouche virus)**	**35 (95)**	**35 (100)**	**21 (60)**	**35 (100)**	**20 (57)**	**31 (89)**	**23 (66)**

EIS: Event Information Site; IHR: International Health Regulations (2005).

^a^ Between 2008 and 2015, all States Parties (*n* = 27) were invited to participate, with the exception of 2014 when only countries who were members of the International Food Safety Authorities Network (INFOSAN) (*n* = 11), a joint initiative of the Food and Agriculture Organization of the United Nations and WHO, were invited. From 2016, all countries and areas in the Western Pacific Region were invited to participate (*n* = 37). No exercise was held in 2009.

^b^ Values are *n*(%).

^c^ Data for IHR (2005) notification and EIS posting completion were not available for 2008 and 2010.

^d^ Dashes indicate that no data were available either because data were missing or the variable was not an objective of the exercise.

The proportion of participating countries and areas that were accessible via e-mail was consistently high, with a mean of 99% for the period 2008–2024 (**Table 3**). However, the proportion of participating countries and areas that were accessible via telephone has been suboptimal, with a mean of 64% for the period of 2008–2024 (**Table 3**). The proportion of participating countries and areas that joined a teleconference or a videoconference (first introduced in 2015) with the Regional Office during the exercise improved over time, with 85% or more successfully attending between 2021 and 2024, for a mean of 73% over the entire period 2008–2024 (**Table 3**).

**Fig. 2:**
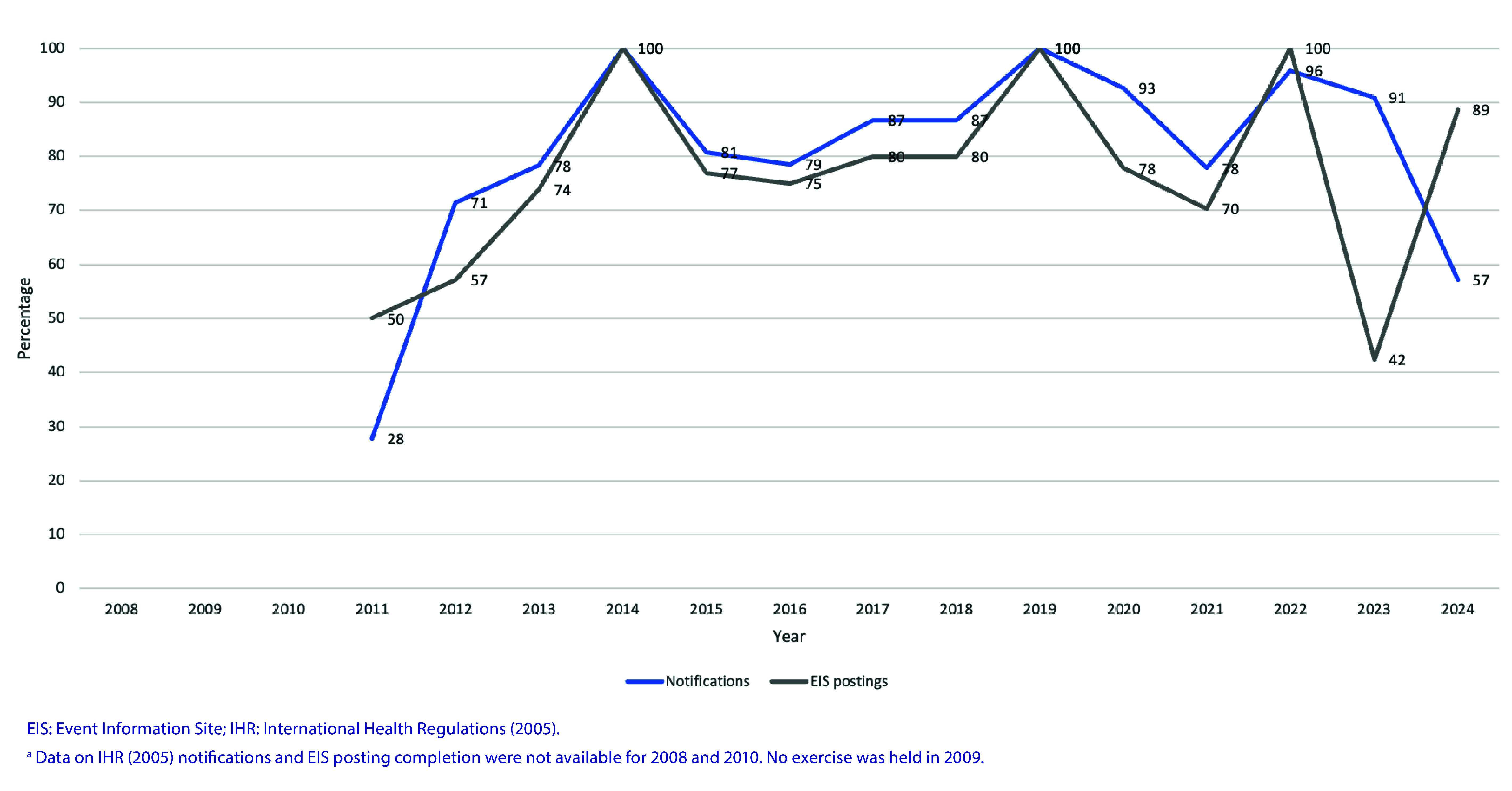
Percentage of participating countries and areas completing IHR (2005) notifications and EIS postings during IHR Exercise Crystal, by year, 2008–2024^a^

The proportion of participating countries and areas notifying the simulated event to the WHO IHR Contact Point has improved over time, with some variation between years. Between 2008 and 2024, a mean of 80% of participating countries and areas notified the simulated event to WHO (**Fig. 2**). The proportion of participating countries and areas that contributed information to an EIS posting has typically followed a similar trend to that of notifications, with some variation in 2023 and 2024. Between 2008 and 2024, a mean of 77% of participating countries and areas contributed information to an EIS posting (**Fig. 2**).

[**insert** Figure 2]

Data for the variable about multisectoral communication were limited for the period 2008–2024, as this was not an objective of IHR Exercise Crystal for 6 of the 16 years (**Table 1**). Although no clear trend in the capacity of NFPs to conduct multisectoral communication was observed over time, the mean performance was 73% for the 9 years of data (**Table 3**). Furthermore, where data were available, the proportion of NFPs engaging in multisectoral communication was lowest for the scenarios based on a radiological event, and vector-borne disease and non-influenza respiratory disease outbreaks caused by *Francisella tularensis* and novel coronavirus, and highest for infectious disease outbreaks involving a novel influenza virus or gastroenteritis (**Table 3**).

## Discussion

Since its beginning in 2008, IHR Exercise Crystal has seen strong participation from Member States in the Western Pacific Region, and several key NFP performance indicators have been tracked over time. Our analysis of these indicators demonstrates that the requirement for 24/7 accessibility of NFPs through their registered contact details continues to be a challenge. E-mail has proven to be the most effective means of communication, with a mean of 99% of NFPs successfully being contacted by e-mail, compared with a mean of 64% of NFPs successfully being contacted by telephone. The proportion of NFPs who are able to join the exercise by tele- or videoconferencing has improved over time. These trends are consistent with previous findings about contacting NFPs in the Region (*12*) and demonstrate that while NFPs’ performance has continued to improve, there remains a need to regularly test and update NFPs’ contact details, at least annually. This is supported by a global survey of States Parties conducted in 2019, which found that communications was one of four critical areas in which NFPs experienced challenges. (*13*)

Similarly, our finding that the proportion of NFPs notifying simulated public health events to the WHO IHR Contact Point and the proportion of NFPs contributing information to EIS postings have improved over time is consistent with findings from other studies. (*12*-*14*) In a study of IHR (2005) States Parties, most (96%) NFPs reported that they were familiar with how to contact their designated WHO IHR Contact Point and that they had the necessary content expertise to discuss a notifiable event with the WHO IHR Contact Point. (*13*) In another study, 88% of NFPs reported that they had excellent or good knowledge of the Annex 2 decision-making instrument and either excellent (23%) or good (44%) ability to assess potential public health emergencies of international concern under Annex 2. (*14*) The strong performance in this NFP capacity is likely due to the long-term investments made by WHO and States Parties in institutionalizing the use of Annex 2 through training, guidance documents and standard operating procedures, as well as the development of legal, regulatory or administrative provisions supporting its use. (*15*-*18*) However, efforts should be continued to reinforce this capacity and ensure that it is consistently applied across all hazards. This need is highlighted by the decrease in EIS submissions during the 2023 Exercise Crystal, which featured a radiological event.

Our analysis did not find a clear trend in NFPs’ capacity to engage in multisectoral communication; however, on average, 73% of NFPs communicated with another sector or agency during exercise play. It had been previously identified that NFPs experience challenges in intersectoral collaboration within their countries, including having limited access to or experiencing a lack of cooperation from key ministries (*13*) and that NFPs were not sufficiently empowered to carry out their functions, (*13*, *19*, *20*) which creates difficulties in engaging directly with other agencies or sectors and in triggering decision-making processes by national health authorities. (*20*) For these reasons, it has been recommended that States Parties establish a National IHR Authority that will focus on implementing the IHR (2005) across sectors at the national level, recognizing that the core capacities of the Regulations extend beyond the health sector. (*20*, *21*) Although the roles and functions of NFPs do not change under the June 2024 amendments, (*3*) the added requirement to designate or establish a National IHR Authority means that it will be even more important to ensure roles and responsibilities are clearly delineated and that States Parties develop, test and maintain up-to-date standard operating procedures, particularly in relation to multisectoral communication and coordination.

States Parties should also continue strengthening and empowering NFPs to conduct their core functions of IHR (2005) communications. Tools such as SPAR and IHR Exercise Crystal can guide continual improvements in NFP functionality, while highlighting the need for a critical foundation of supporting legislation and sufficient resourcing. (*22*) Furthermore, APHSAF advocates for strengthening the mandate and capacities of NFPs, by ensuring that they are prepared and ready to respond to public health emergencies (e.g. through regular testing), and by enhancing communication, information-sharing and coordination between the National IHR Focal Point system and emergency contacts for other areas and sectors, as well as between countries. (*4*)

Limitations of this analysis include the inability to capture multisectoral communications that occur outside of e-mail communications observed during an exercise and variations over time in the methods used for monitoring and evaluating IHR Exercise Crystal. Therefore, it is important to consider triangulation with multiple data sources, such as SPAR, when analysing and interpreting the results of simulation exercises. Furthermore, as this study measured IHR (2005) communications in a simulation setting, actual NFP performance in real events may vary due to real-world complexities not reflected in the exercises.

### Conclusions

Between 2008 and 2024, States Parties in the WHO Western Pacific Region demonstrated improved NFP capacities in the areas of IHR (2005) notification, contributing information to EIS postings, and participating in tele- and videoconferencing. Continued strengthening is required, particularly in the areas of NFP accessibility and multisectoral communications, alongside ongoing efforts to standardize data collection and assessments. Simulation exercises such as IHR Exercise Crystal are one tool that States Parties can use to assess NFP capacities and guide improvements. NFP functions do not change in the context of the IHR (2005) amendments and the designation or establishment of a National IHR Authority; however, States Parties should clearly define each entity’s responsibilities, and develop and test operational procedures to ensure that NFPs continue to function without disruption. This is critical to advancing health security and IHR (2005) implementation in the Region.
